# SSNFNet: An Enhanced Few-Shot Learning Model for Efficient Poultry Farming Detection

**DOI:** 10.3390/ani15152252

**Published:** 2025-07-31

**Authors:** Bingli Wang, Daixian Liu, Jinghua Wu

**Affiliations:** College of Information Engineering, Sichuan Agricultural University, Ya’an 625014, China; wangbingli0602@foxmail.com (B.W.);

**Keywords:** few-shot learning, poultry farming, object detection, artificial intelligence, optimization algorithm

## Abstract

Smart agriculture addresses inefficient resource utilization and disease control in poultry farming. Existing smart monitoring systems effectively detect birds. However, applying these models to new environments or for detecting new species requires a large amount of labeled data and manual work, which limits their wide application. To address this limitation, this paper presents the SSNFNet method, leveraging an enhanced few-shot object detection framework tailored for poultry farming contexts. SSNFNet integrates Sharpness-Aware Minimization (SAM) to enhance model generalization by smoothing the loss landscape and improving training stability. To further improve detection in densely populated scenes, we incorporate the Soft Non-Maximum Suppression (Soft-NMS) algorithm to mitigate overlapping bounding box issues. Through quantitative analysis and comparison, exemplified by a five-shot scenario on the poultry farming dataset, our method demonstrates significantly better performance compared to traditional object detection models, Specifically, it achieves a mean Average Precision (mAP) improvement of 3.93% compared to the state-of-the-art HTRPN model, raising the mAP from 78.00% to 81.93% while maintaining 8 FPS inference speed on Jetson Nano-class hardware. These results confirm the effectiveness and adaptability of our approach in real-world smart farming environments.

## Simple Summary

Poultry farming struggles with inefficiency and disease control, while existing smart monitoring systems require large amounts of labeled data to detect new types, limiting their practical use. To address this, we developed SSNFNet, a method that works effectively with only a few training images, making it easier to adapt to different farms. Our approach improves detection in crowded environments where birds often overlap, and performs well even with new species. We trained the model mostly on ducks and then used a small number of images of chickens to teach it to recognize them, demonstrating its ability to learn quickly. We also tested it on goldfish to confirm its flexibility. Results show that our method outperforms standard detection systems and other few-shot learning models by 3.93%. Key improvements include Sharpness-Aware Minimization (SAM), which stabilizes training with limited data, and Soft-NMS, which reduces errors when detecting birds in dense groups. This technology helps farmers monitor poultry more accurately with less manual effort, making smart farming more accessible, especially where collecting large datasets is difficult. By improving efficiency and reducing reliance on extensive labeling, our method supports sustainable and cost-effective poultry farming.

## 1. Introduction

As the public increasingly focuses on healthy diets, protein-rich foods have become their preferred choice, and poultry products ideally meet this demand. Not only is poultry rich in protein, but it is also relatively low in fat, making it a widely regarded nutritionally balanced food option that has won growing consumer favor. Data from 2022 indicate that China’s poultry output reached 16.14 billion units, a year-on-year increase of 400 million with 2.5% growth. The country’s poultry-meat production reached 24.43 million tons (up 630,000 tons or 2.6%), while egg production stood at 34.56 million tons (a rise of 480,000 tons or 1.4%). As of year-end 2022, China’s live poultry inventory totaled 6.77 billion, recording a slight 0.2% decrease year-on-year [1]. These figures demonstrate the poultry industry’s expansion scale and its development momentum in adapting to market demand.

As an integral part of the agricultural sector, traditional poultry farming has long provided us with abundant food resources and significant economic benefits [2]. However, it also faces serious challenges and limitations that cannot be ignored. In this conventional breeding model, the entire process relies heavily on the personal experience and subjective judgment of farmers, lacking scientific management and meticulous operation, which inevitably leads to waste of resources and low production efficiency. Taking the use of feed as an example, its often imprecise control leads to unnecessary waste, not only increasing the cost of breeding, but also imposing an additional burden on the environment [3]. More importantly, such feed mismanagement can create unhygienic conditions in poultry houses, as spilled feed attracts pests and promotes bacterial growth. This hygiene issue directly correlates with disease prevention challenges in poultry farming [4]. Currently, the detection of poultry diseases mainly relies on manual observation, which is prone to misdiagnosis or missed diagnosis, and the unsanitary conditions caused by feed spillage may further exacerbate disease risks. These challenges in production not only reduce farm efficiency, but also directly impact the safety and quality of poultry products reaching consumers. For instance, poor hygiene and disease outbreaks can lead to contaminated meat or eggs, while the lack of precise monitoring makes it difficult to ensure proper traceability. As a result, as consumers’ expectations for food safety and quality continue to rise, the traditional poultry farming model is no longer able to meet the market demand for high-quality, traceable products.

Smart agriculture has perfectly integrated advanced information and communication technology with modern agricultural equipment, significantly elevating the level of automation and intelligence in agricultural production and effectively overcoming numerous challenges faced in traditional animal husbandry. By deploying environmental monitoring devices, such as temperature and humidity sensors and ammonia detectors, we are able to monitor the breeding environment in real-time, ensuring that the conditions necessary for the growth of farm animals are optimal [5]. Furthermore, by attaching electronic tags to the animals, we can record and track the growth indicators and environmental factors of the animals in real-time [6]. Consumers can quickly access comprehensive information about the animals, from birth to slaughter, by simply scanning the relevant barcodes or QR codes. Behind all these advancements, artificial intelligence plays a central role in driving the continuous progress of smart agriculture, laying a solid foundation for in-depth research and widespread application in the field of animal husbandry.

The rapid advancement of artificial intelligence has become a hot topic in the field of technology in recent years, particularly with the rise of deep learning and the widespread application of neural networks. In the last few years, researchers have achieved groundbreaking results in natural language processing (NLP) using deep learning techniques [7]. For instance, pre-trained models such as BERT, GPT-4, and T5 have demonstrated impressive performance across various NLP tasks, including text generation, machine translation, and question-answering systems [8,9,10]. In computer vision tasks, such as object detection with the YOLO series, multiple objects in images can be efficiently detected, playing a crucial role in industrial automation, traffic supervision, and autonomous driving [11]. Additionally, semantic segmentation is another area garnering attention, involving the assignment of each pixel in an image to a specific semantic category. Pre-trained convolutional neural networks like DeepLab, UNet, and SegNet have driven performance improvements in semantic segmentation tasks, showcasing broad application prospects in fields such as medical image analysis and urban planning [12,13,14].Recently, Umirzakova et al. [15] proposed MIRA-CAP (Memory-Integrated Retrieval-Augmented Captioning), a novel framework that enhances both image and video captioning by leveraging a cross-modal memory bank, adaptive dataset pruning, and a streaming decoder, achieving state-of-the-art performance on standard datasets.

While artificial intelligence is rapidly advancing, its application in smart agriculture largely remains in the research phase. Dihua Wu [16] utilized an improved ResNet-50 deep learning algorithm to identify the gender of chickens, addressing the inefficiencies associated with traditional manual observation. Recent studies have further demonstrated the potential of deep learning in poultry behavior monitoring and farm management. Paneru et al. [17] developed specialized YOLOv8 models for automated detection of dustbathing behavior in cage-free laying hens, achieving over 93% precision in tracking this welfare-related behavior across different growth phases. Similarly, Yang et al. [18] proposed an innovative Depth Anything Model (DAM) that enables accurate poultry drinking behavior monitoring and floor egg detection using only RGB images, with 92.3% accuracy in behavior recognition and significant improvements in farm operation efficiency.

In the field of poultry farming, although traditional object detection technologies have made some progress, they still have many limitations. Firstly, poultry present a high degree of diversity and complexity in actual breeding environments [19]. This results in the models often exhibiting insufficient generalization ability when dealing with different types, environments, and species of poultry, which increases the difficulty of operation and maintenance. Secondly, traditional object detection methods typically require a large amount of annotated data for training models, which is challenging and expensive to obtain in real poultry farming scenarios, potentially failing to meet high requirements for real-time performance [20]. Furthermore, for some rare breeds, it is particularly difficult to obtain enough data samples due to their limited number and the restrictions on protection and management. Finding an object detection method that can maintain high performance under conditions of scarce samples is of significant importance for enhancing the level of intelligence in poultry farming.

Few-shot learning has emerged as a crucial research direction in the field of machine learning, aiming to address the significant performance degradation traditional machine learning methods experience when faced with scarce data [21]. Particularly in specialized domains like healthcare and agriculture, the acquisition of a substantial amount of labeled data can be time-consuming and expensive, underscoring the prominence of this challenge. Few-shot learning innovates by designing more efficient algorithms and models, enabling machine learning models to perform well even when only a limited number of samples are available. This approach substantially reduces the complexity and cost associated with data preparation and enhances the model’s generalization capabilities, especially when encountering unseen categories or scenarios. With the rapid advancements in deep learning technologies, few-shot learning has made remarkable progress in various fields, such as image recognition, segmentation tasks, and speech recognition, in recent years, becoming a significant driving force behind the continuous innovation in artificial intelligence technologies [22,23,24].

While few-shot learning has shown promising results in various vision tasks, its performance in agricultural settings—particularly poultry detection—remains suboptimal. This is primarily due to the unique challenges in such environments, including high intra-class variance among birds (e.g., differences in breed, size, and plumage), crowded scenes with frequent occlusions, and significant variations in lighting and background conditions. Most existing FSOD methods are designed for general object categories and fail to account for these domain-specific complexities. As a result, there is a clear need for tailored solutions that can better adapt to the visual diversity and environmental variability inherent in agricultural monitoring tasks.

To overcome the challenges encountered in practical applications, this paper integrates few-shot object detection with poultry farming. As a novel paradigm in object detection, few-shot object detection can achieve accurate detection and recognition of poultry with only a small number of annotated samples, effectively addressing the complexity of the breeding environment and the diversity of animal species [25]. By just using between one and five images, few-shot object detection can fulfill the task of detecting poultry, circumventing the need for large-scale datasets, saving time and resources in data collection and annotation, and enhancing the model’s generalization capabilities and robustness. This paper innovatively applies the few-shot object detection technique to two common types of poultry, chickens and ducks, using ducks as the base class model for training. Subsequently, chickens are introduced as a new class for few-sample learning and testing. To demonstrate the model’s generalization ability, goldfish are also tested as a new class. The ultimate goal is to achieve efficient and accurate animal detection with a limited number of samples.

This study employs the FSCE (Few-Shot Object Detection via Contrastive Proposal Encoding) as its foundational model, an innovative approach tailored for few-shot object detection [26]. However, in the nuanced domain of poultry farming, characterized by diverse terrains, variable climates, and myriad breeding practices, object detection presents significant challenges. The propensity of these animals to congregate in dense clusters exacerbates the issue, as standard object detection methods struggle with multiple overlapping detection boxes against a backdrop of highly variable environmental conditions. This complexity underscores the need for a robust model capable of navigating the unique challenges posed by the poultry farming landscape.

To significantly improve the performance and stability of our model in the intricate settings of poultry farming, we have integrated the advanced Sharpness-Aware Minimization (SAM) technique into our framework [27]. SAM refines the optimization process by directly targeting the smoothness irregularities within the loss function. This strategic approach ensures a more stable model convergence, especially critical in environments with complex agricultural backgrounds and densely populated animal scenes.

Furthermore, recognizing the unique challenge posed by the tendency of poultry to cluster, resulting in numerous overlapping detection boxes, we have innovatively adopted the Soft-NMS algorithm as a pivotal enhancement to our model. Traditional Non-Maximum Suppression (NMS) methods, while effective in certain contexts, fall short in densely populated scenarios by indiscriminately discarding all but the highest-scoring overlapping boxes. This often leads to the erroneous elimination of valid detections [28]. In contrast, Soft-NMS employs a more nuanced approach [29]. By decrementing the scores of overlapping boxes rather than outright exclusion, Soft-NMS substantially improves the retention of accurate detection boxes for partially obscured animals. This nuanced adaptation not only addresses the inherent limitations of traditional NMS in complex detection scenarios, but also markedly enhances our model’s capability to accurately identify poultry.

In summary, the contributions of this paper are as follows:

- We have innovatively introduced few-shot object detection technology into smart agriculture, aiming to enhance the practical performance of models in poultry farming.
- To address the issue of model performance fluctuations caused by non-smooth loss in poultry farming environments, we have incorporated the Sharpness-Aware Minimization (SAM) model. This optimization enhances the smoothness of the loss function during model training, leading to improved stability and accuracy of the model in complex backgrounds.
- To tackle the challenges posed by the high-density (average occlusion rate > 10%) congregation of farm animals, we have adopted Soft-NMS as a replacement for the traditional NMS method, reducing the wrongful deletion of occluded targets and significantly improving the model’s detection performance in dense scenarios.

## 2. Materials and Methods

### 2.1. Dataset Collection

In this study, ducks and chickens, being the most common poultry species, were selected as the primary subjects of research. To demonstrate the model’s generalization and applicability, fish were also included as subjects of study. This selection is based on several key factors: Firstly, these species are ubiquitous in agricultural breeding, representing a wide range of breeding environments and challenges. Secondly, the behavioral and physiological differences between ducks, chickens, and fish provide a rich diversity of data, aiding in the verification and enhancement of the object detection model’s generalization capability. Additionally, these species hold significant positions in the agricultural economy, making their study valuable for improving agricultural production efficiency and animal welfare. Therefore, this research aims to provide more precise and efficient object detection solutions for the agricultural breeding sector through an in-depth analysis of these species.

**Figure 1:**
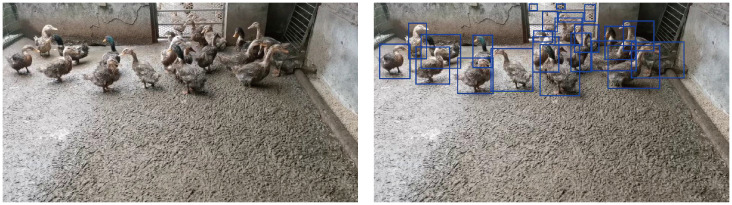
This figure is divided into two sections. (**Left**): Showcasing the original duck breeding dataset image. (**Right**): A labeled diagrammatic representation of the dataset.

The duck dataset used in this study specifically selected the hemp duck breed [30]. Hemp ducks are not only abundant and widely distributed, but also display unique biological characteristics and behavioral patterns. In this project, hemp ducks were raised in a high-density captive environment, presenting significant challenges in distinguishing adjacent individuals and addressing issues of visual obstruction. These characteristics of hemp ducks not only provided a rich dataset for this study, but were also instrumental in testing and enhancing the accuracy and generalizability of the SSNFNet model in complex rearing environments. An image of a hemp duck is as shown in Figure 1.

**Figure 2:**
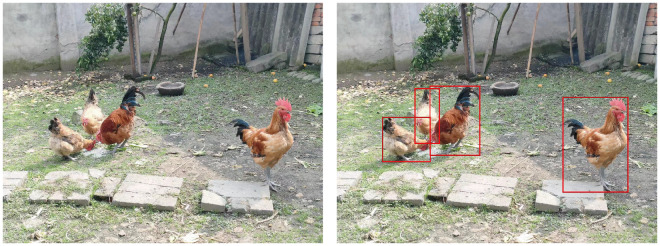
This figure is divided into two sections. (**Left**): Showcasing the original chicken breeding dataset image. (**Right**): A labeled diagrammatic representation of the dataset.

The dataset of chickens for this research was meticulously gathered via on-site surveys by our project members, primarily sourced from several family-owned poultry farms around Pengzhou City, Chengdu, Sichuan Province, China. Representations of these chickens are illustrated in Figure 2. These datasets encompass a variety of family farming environments, including natural settings with muddy grounds and tree cover, as well as smoother cement surfaces with minimal obstructions. They offer a rich, authentic, and diverse perspective for research, capturing chickens in various environments and their postures during daily activities such as foraging, walking, and resting. Additionally, special attention was paid to variations in lighting conditions and shooting distances to enhance the dataset’s quality and diversity, ensuring the collection of more comprehensive and realistic data. Through this meticulous data collection approach, the study aims to provide a thorough and high-quality training foundation for the SSNFNet model.

**Figure 3:**
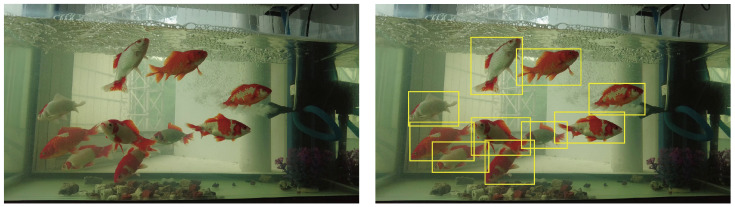
This figure is divided into two sections. (**Left**): Showcasing the original fish breeding dataset image. (**Right**): A labeled diagrammatic representation of the dataset.

To comprehensively evaluate the robustness and scalability of the SSNFNet model, this study has specifically incorporated a dataset of aquatic life, focusing on fish, for in-depth testing. The fish dataset centers on the golden crucian carp, which is an ancestral species of the goldfish [31]. Images capturing these fish are presented in Figure 3. The lifespan of the golden crucian carp is significantly influenced by temperature, with susceptibility to diseases increasing beyond optimal temperature ranges. Fish, compared to the previously discussed ducks and chickens, exhibit significant differences in morphology, behavior, and habitat. Their unique underwater environment introduces new challenges to object detection, such as underwater blurriness and light refraction. The successful application of the SSNFNet model on the fish dataset not only validates its effectiveness in poultry farming, but also demonstrates its potential applicability in aquaculture, showcasing its adaptability and robustness in complex environments.

### 2.2. Method

#### 2.2.1. Dataset Production

To ensure the quality and diversity of our data, meticulous manual screening was conducted to eliminate highly redundant data, preventing overfitting during the model training phase. This step is crucial for maintaining the authenticity and effectiveness of our dataset. The dataset annotation was carried out by four members of our project team, using LabelImg, a popular image annotation tool that enables users to mark the location and category of target objects within images. Throughout the annotation process, each team member strictly adhered to consistent standards, ensuring the accuracy and uniformity of the annotations. Such thorough data processing and annotation practices allow us to provide a high-quality, information-rich dataset for training the SSNFNet model. These meticulously prepared datasets will assist the model in better learning and recognizing target species across various environments, thereby enhancing its accuracy and generalizability in practical applications.

#### 2.2.2. Model Architecture of SSNFNet

SSNFNet incorporates the FSCE (Few-Shot Object Detection via Contrastive Proposal Encoding) model as its core for pioneering few-shot object detection within the domain of poultry farming [26]. Through contrastive learning, SSNFNet effectively narrows the gap between objects of the same class while widening the distinction between different classes, thereby significantly enhancing detection accuracy in scenarios with extremely limited samples. During the fine-tuning phase, the model locks the feature extraction capabilities of the backbone network to preserve previously acquired knowledge, while adjustments in the Feature Pyramid Network (FPN) and Region Proposal Network (RPN) are permitted to bolster adaptability to new categories. SSNFNet inherits FSCE’s Contrastive Proposal Encoding (CPE) loss integrated into the Region of Interest (RoI) feature extraction process of the Faster RCNN. This loss is jointly optimized with traditional classification and regression losses through a multi-task learning approach, aiming to minimize the misclassification of instances of new categories as easily confused categories.

**Figure 4:**
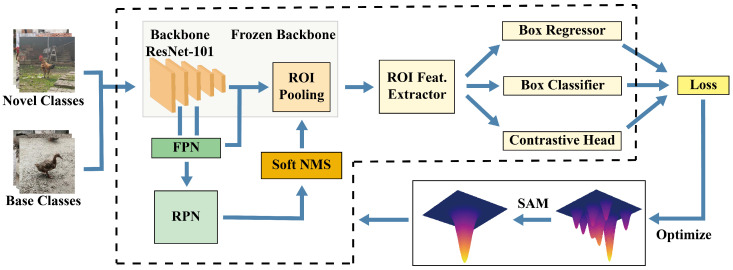
This diagram illustrates the network architecture of SSNFNet, which employs a two-stage fine-tuning approach. Following the RPN, Soft-NMS is introduced, significantly enhancing its accuracy in identifying and distinguishing overlapping objects. Additionally, the integration of Sharpness-Aware Minimization during the training process guides the model towards flatter minima, optimizing its generalization capabilities. These features make SSNFNet particularly well-suited for the complex scenarios typically encountered in poultry farming environments.

The network architecture of SSNFNet is depicted in Figure 4. SSNFNet adopts a two-stage training approach, initially utilizing Faster R-CNN [32] as its foundational detection model with ResNet-101 [33] serving as the backbone to extract image features. Subsequently, it leverages the Feature Pyramid Network (FPN) to generate feature maps of varying scales for detecting objects of different sizes. The Region Proposal Network (RPN) then generates candidate regions on these feature maps. Following this, ROI Pooling extracts fixed-size feature representations from these candidate regions, culminating in the prediction of each region’s category and its precise location through classification and bounding box regression. Unlike the traditional two-stage fine-tuning approach, which freezes the feature extractor components during the new fine-tuning phase and only fine-tunes the box predictor on a balanced subset including both base and novel classes [34], SSNFNet introduces a novel fine-tuning strategy. In this tailored phase for SSNFNet, the adaptability of the Region Proposal Network (RPN) and Regions of Interest (ROI) is preserved, enabling dynamic adjustments to new data inputs. Notably, SSNFNet modifies the Non-Maximum Suppression (NMS) in the RPN to Soft-NMS, offering a more flexible approach to handling overlapping regions, especially in densely populated poultry scenarios. Furthermore, SSNFNet employs Sharpness-Aware Minimization (SAM) to seek flatter minima, thereby enhancing the model’s generalization capabilities.

A pivotal factor in choosing FSCE as the base for SSNFNet is its innovative method for encoding object proposals. Where traditional object detection methods typically struggle with limited sample sizes, FSCE counters this by incorporating a contrastive learning mechanism, significantly boosting the model’s capability to distinguish between objects on sparse data. Specifically, the model is trained by calculating the semantic similarity between different object proposals, encouraging the learned features to be more compact within the same category and more dispersed across different categories. FSCE adds a contrastive branch to the primary Region of Interest (RoI) head. Since RoI features cannot directly calculate the feature similarity between two proposals, the contrastive branch employs a one-layer multi-layer perceptron (MLP) head to encode RoI features into contrastive features $z\in R^{D_{C}}$, with a default $D_{C}=$ 128. Subsequently, the similarity of object proposals is assessed using the MLP-head encoded RoI features, enhancing the consistency of proposals within the same category and the distinctiveness of proposals from different categories through a contrastive objective.

FSCE employs a cosine similarity-based bounding box classifier. In this framework, the prediction of the *i*-th instance belonging to the *j*-th class is determined by calculating the scaled cosine similarity between the RoI feature $x_{i}$ and the class weight $w_{j}$ on a hypersphere,

(1) $$logit_{\left\{i,j\right\}}=\alpha \frac{x_{i}^{⊤}w_{j}}{\|\;x_{i}\;\left\|\;\cdot \;\right\|\;w_{j}\;\|}$$

incorporating a scaling factor α to enhance the gradient (set to 20 in experiments). The contrastive branch in FSCE endows the RoI head with the capability to learn contrastive features that aid in distinguishing between different categories. These features, projected onto the cosine hypersphere, form tight clusters with significant distances between them, effectively improving the model’s generalizability in a few-shot learning scenario.

Furthermore, the FSCE model introduces an innovative loss function, termed the Contrastive Proposal Encoding Loss (CPE Loss), aimed at further enhancing the model’s performance. Specifically, this loss function deals with a mini-batch of *N* RoI box features ${\left\{z_{i},u_{i},y_{i}\right\}}_{i=1}^{N}$, where $z_{i}$ represents the contrastive head encoded RoI feature of the *i*-th region proposal, $u_{i}$ is the Intersection-over-Union (IoU) score with its matched ground truth bounding box, and $y_{i}$ is the corresponding ground truth label,

(2) $$L_{CPE}=\frac{1}{N}\sum_{i=1}^{N}f\left(u_{i}\right)\cdot L_{z_{i}}$$

(3) $$L_{z_{i}}=\frac{-1}{N_{y_{i}}-1}\sum_{j=1,j\neq i}^{N}I\left\{y_{i}=y_{j}\right\}\cdot \log\frac{\exp({\tilde{z}}_{i}\cdot {\tilde{z}}_{j}/\tau )}{\sum_{\begin{array}{c} k=1 \end{array}}^{N}I_{k\neq i}\cdot \exp\left({\tilde{z}}_{i}\cdot {\tilde{z}}_{k}/\tau \right)}$$

$N_{y_{i}}$ represents the number of proposals with the same label as $y_{i}$, and τ is the hyperparameter representing temperature. $\tilde{z}i=\frac{zi}{\left|z_{i}\right|}$ signifies the normalized features, thereby $\tilde{z}i\cdot \tilde{z}j$ measures the cosine similarity between the *i*-th and *j*-th proposals in the projected hypersphere. By enhancing the intra-class compactness and inter-class distinctiveness at the instance level, the CPE loss function effectively reduces the misclassification of new class instances as easily confused categories.

In the FSCE model, a novel approach is adopted for proposal consistency control. Unlike image classification, which relies on the entire image, detection hinges on classification signals from region proposals. FSCE introduces an IoU threshold to ensure the consistency of proposals used for contrastive purposes. This approach is based on the premise that proposals with low IoU might deviate significantly from the center of regressed objects, potentially containing irrelevant semantic information. Within this framework, the function $f(u_{i})$ is employed to maintain proposal consistency, defined by the proposal consistency threshold ϕ and a re-weighting function g(·),

(4) $$f\left(u_{i}\right)=I\left\{u_{i}\geq \phi \right\}\cdot g\left(u_{i}\right)$$

specifically, g(·) assigns varying weight coefficients to object proposals based on different IoU levels. It has been observed that a threshold of ϕ=0.7 effectively trains the contrastive head with the most centrally aligned object proposals.

In the first stage, the base detector is trained with the standard losses of Faster R-CNN [32]. This includes binary cross-entropy loss $L_{rpn}$ for foreground proposal generation, cross-entropy loss $L_{cls}$ for bounding box classification, and smooth-*L*1 loss $L_{reg}$ for box regression. In the fine-tuning stage for novel data, the training incorporates a contrastive loss combined with the primary Faster R-CNN loss in a multi-task approach, ensuring stable training,

(5) $$L=L_{rpn}+L_{cls}+L_{reg}+\lambda L_{CPE}$$

λ is set at 0.5 to balance the scale of the losses.

#### 2.2.3. Soft-NMS

Non-Maximum Suppression (NMS) plays a crucial role in object detection, primarily functioning to reduce false positives by eliminating redundant bounding boxes. In the traditional NMS approach, bounding boxes are sorted by their scores, selecting the highest-scoring box (M) as the primary one and removing all others that overlap beyond a certain threshold [28]. This method can inadvertently lead to the loss of valid targets in scenarios with highly overlapping objects. In contrast, Soft-NMS adopts a more flexible approach, where it does not immediately discard bounding boxes with high overlap [29]. Instead, it reduces their scores proportionally to the degree of overlap. This means that even if there is some degree of overlap between bounding boxes, they may still be retained, albeit at a lower priority.

In densely populated scenarios, such as agricultural breeding environments, animals like chickens, ducks, and fish are often closely packed or partially overlapping. In these situations, traditional Non-Maximum Suppression (NMS) struggles to differentiate between adjacent individuals [28]. By directly eliminating bounding boxes with high overlap, traditional NMS might miss key targets in agricultural settings, leading to lower overall detection accuracy. In contrast, Soft-NMS stands out for its flexibility in handling overlapping detections [29]. By reducing excessive suppression in overlapping areas, it adapts more effectively to animals of various types and sizes. Even in situations where animals are closely packed or partially overlapping, Soft-NMS can more accurately identify and distinguish each individual, thereby enhancing object detection accuracy in agricultural environments.

**Figure 5:**
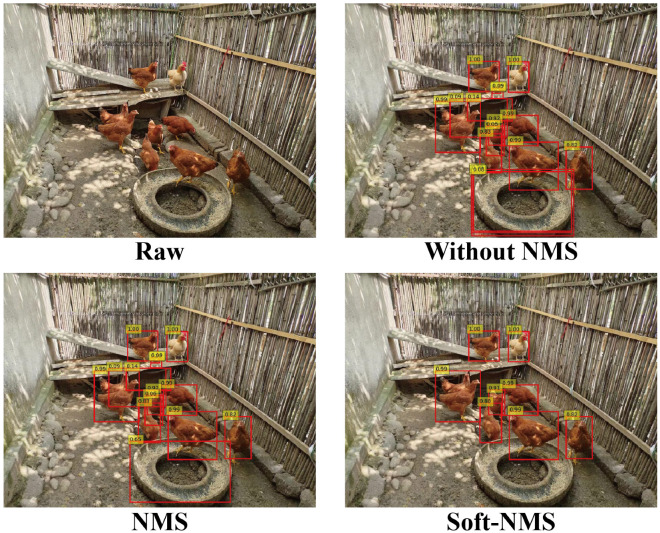
The (**top left**) corner shows the original image of the chicken flock. The (**top right**) corner displays bounding boxes before any Non-Maximum Suppression processing. The (**bottom left**) corner presents the bounding boxes after traditional NMS processing. The (**bottom right**) corner illustrates the bounding boxes processed with Soft-NMS.

As shown in Figure 5, the top left corner displays an original image of chickens in an agricultural farming environment. This image captures the natural behaviors of chickens in a typical farming setting, such as pecking and walking. The top right corner shows the detection boxes before Non-Maximum Suppression (NMS) processing, which include multiple rectangular boxes marked by the object detection algorithm on the original image to indicate the positions of the chickens. These detection boxes, not having undergone any suppression, may overlap, showing all potential detection outcomes. The bottom left corner presents the detection boxes after traditional NMS processing, where only the highest-scoring box for each chicken is retained, reducing overlap, but potentially missing some chickens. The bottom right corner illustrates the detection boxes processed with Soft-NMS, which accurately identifies each chicken even in situations of occlusion.

The traditional Non-Maximum Suppression (NMS) algorithm determines which bounding boxes should be retained by comparing their overlap. This process is primarily implemented through a pruning step, mathematically expressed as follows:

(6) $$s_{i}=\left\{\begin{array}{cc} s_{i}, & \mathrm{IoU}\left(M,b_{i}\right)<N_{t}\\ 0, & \mathrm{IoU}\left(M,b_{i}\right)\geq N_{t} \end{array}\right.,$$

$s_{i}$ represents the score of the *i*-th bounding box. $\mathrm{IoU}(M,b_{i})$ denotes the Intersection over Union (IoU) between two bounding boxes, a metric for assessing the extent of overlap. Here, M represents the bounding box with the highest score and $b_{i}$ is the *i*-th bounding box. $N_{t}$ is the IoU threshold. If the IoU of the bounding box $b_{i}$ with M is less than $N_{t}$, the original score $s_{i}$ is retained. Otherwise, if the IoU exceeds $N_{t}$, the score is set to 0, effectively removing the bounding box from the set considered in object detection tasks.

The traditional Non-Maximum Suppression (NMS) method, which zeroes out the scores of bounding boxes below a certain threshold, can be too rigid. In contrast, Soft-NMS adopts a more flexible approach by dynamically adjusting the scores based on the Intersection over Union (IoU) between bounding boxes, thus reducing the wrongful deletion of valid targets. Soft-NMS proposes two methods of score decay.

The first is a linear penalty function, where the scores of detections above the threshold $N_{t}$ are linearly decayed based on their overlap with the highest scoring box M. This means that boxes with less overlap with M are less affected, while those with higher overlap receive greater penalties. The function is as follows:

(7) $$s_{i}=\left\{\begin{array}{cc} s_{i}, & \mathrm{IoU}\left(M,b_{i}\right)<N_{t}\\ s_{i}\left(1-\mathrm{IoU}\left(M,b_{i}\right)\right), & \mathrm{IoU}\left(M,b_{i}\right)\geq N_{t} \end{array}\right..$$

The second method is a Gaussian penalty function, addressing the issue of score ranking disruptions when IoU values are slightly below or above the threshold. The Gaussian function assigns higher penalties to boxes with higher IoU and lower penalties to those with lower IoU, with a smooth transition between the two. This approach effectively avoids issues related to threshold settings.Updating the pruning step using a Gaussian penalty function can be performed as follows:

(8) $$s_{i}=s_{i}e^{-\frac{\mathrm{IoU}{\left(M,b_{i}\right)}^{2}}{\sigma }},\forall b_{i}\notin D.$$

The Soft-NMS algorithm, as illustrated in Algorithm 1, begins by sorting all detected bounding boxes based on their confidence scores, selecting the highest-scoring box as the reference. For each remaining bounding box, the Intersection over Union (IoU) with the reference box is calculated to assess the degree of overlap. Boxes that overlap with the reference box beyond a preset threshold are not outright deleted, but have their confidence scores reduced according to the degree of overlap. Subsequently, the highest-scoring box from the remaining ones is chosen as the new reference, and the process is repeated until all boxes have been processed. Ultimately, all boxes that have not been deleted and whose scores have not fallen below the threshold are considered the final detection results.
**Algorithm 1** Soft-NMS
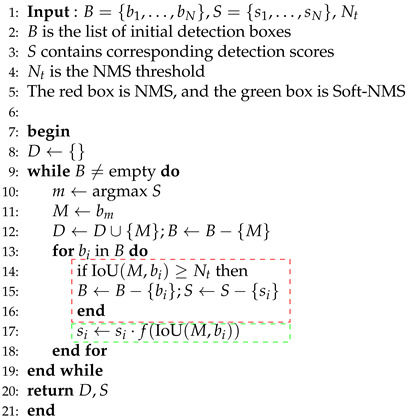


#### 2.2.4. Sharpness-Aware Minimization

In agricultural breeding, extreme environmental variability—including diverse lighting conditions, complex animal behaviors, and species diversity—poses major challenges for precise target detection. These factors complicate learning from limited samples and accurate prediction, especially when visually similar species coexist. Achieving high detection accuracy in such high-variability, low-data scenarios remains particularly difficult.

To address these challenges, we adopt Sharpness-Aware Minimization (SAM) [27], which enhances model generalization by seeking flat low-loss regions in parameter space. This approach reduces overfitting to specific training samples, encouraging the model to learn generalizable features from scarce data. SAM-trained models show improved accuracy in identifying animals against complex backgrounds, offering critical support for high-precision detection in agricultural settings.

**Figure 6:**
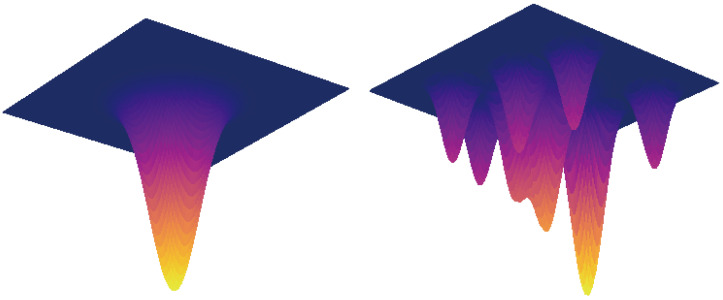
The graph on the (**left**) is the convergence to the wide minimum, and the (**right**) is the convergence to the sharp minimum.

SAM, as shown in Figure 6, minimizes both the loss value and its local sensitivity (sharpness), where sharpness quantifies how the loss fluctuates with parameter perturbations. High sharpness indicates that dramatic loss increases under small changes, while low sharpness implies stability. In overparameterized models, minimizing training loss alone often leads to poor generalization, as flat minima (stable regions) are theoretically preferable to sharp minima (sensitive regions).

Flat minima exhibit lower sensitivity to input variations, producing more consistent predictions compared to sharp minima. SAM optimizes parameters to achieve low loss values across entire neighborhoods rather than isolated points, prioritizing both low loss and flatness. This dual focus theoretically ensures its superior generalization capabilities for real-world applications.

The Sharpness-Aware Minimization (SAM) algorithm addresses generalization by minimizing the worst-case loss within a neighborhood around parameters *w*. Starting from inequality:

$$L_{D}\left(w\right)\leq \max_{{∥\epsilon ∥}_{2}\leq \rho }L_{S}\left(w+\epsilon \right)+\lambda {∥w∥}_{2}^{2},$$

where the key idea is to optimize both the empirical risk $L_{S}$ and its sensitivity to perturbations ϵ, controlled by the neighborhood size ρ and weight decay λ.

Instead of directly solving the full minimax problem, SAM approximates the worst-case parameter perturbation $\epsilon^{*}\left(w\right)$ by linearizing $L_{S}$ around *w*:

$$\epsilon^{*}\left(w\right)\approx \arg\;\max\;{∥\epsilon ∥}_{p}\leq \rho \;\epsilon^{⊤}\nabla_{w}L_{S}\left(w\right).$$

This simplification transforms the inner maximization into a dual norm problem, which admits a closed-form solution when 1/p+1/q=1. Intuitively, this step identifies the direction in parameter space that most rapidly increases the loss.

The final gradient update combines the original loss gradient with this adversarial perturbation. Practically, SAM computes

$$\nabla_{w}L_{S}^{\mathrm{SAM}}\left(w\right)\approx \nabla_{w}L_{S}\left(w+\hat{\epsilon }\left(w\right)\right),$$

where $\hat{\epsilon }\left(w\right)$ is the normalized perturbation derived from the dual norm. This approximation avoids second-order terms, making SAM computationally efficient while still encouraging flat minima that improve generalization.

Empirically, setting p=2 (Euclidean norm) often yields optimal performance, as it balances robustness to perturbations with geometric interpretability in high-dimensional spaces.

#### 2.2.5. Summary of Methodological Contributions

Our SSNFNet model achieves superior few-shot object detection in challenging poultry farming environments by addressing the key limitations of other methods through contrastive learning, Soft-NMS, and Sharpness-Aware Minimization (SAM). To tackle the issue of intra-class variance, SSNFNet incorporates a Contrastive Proposal Encoding (CPE) loss, which enhances feature compactness within the same class while increasing inter-class separability. This enables the model to better distinguish between visually similar instances, even under significant appearance variations commonly found in real-world agricultural scenes. The CPE loss, together with cosine similarity-based classification, helps form tight and well-separated feature clusters, thereby improving generalization from limited samples.

In addition, Soft-NMS improves performance in dense, crowded scenes by gracefully reducing scores of overlapping boxes instead of discarding them, crucial for preserving detections and enhancing recall with limited data. SAM further enhances robustness and generalization from scarce, noisy data by seeking flat loss minima, avoiding the sharp, noise-sensitive minima that can destabilize few-shot training.

## 3. Results

### 3.1. Experiments Setting

In this study, the SSNFNet model is trained and validated on the poultry dataset. The experimental setup designates ducks as the base class, with chickens and fish considered as novel categories for research. Additionally, the fish dataset is specifically chosen to represent aquatic life, testing the robustness of the SSNFNet model in diverse environments. The dataset consists of 500 images of ducks and 60 images each of chickens and fish. We use Faster-RCNN [32] with Resnet-101 [33] and Feature Pyramid Network [35] as the detection model. The training of the SSNFNet model is conducted in two stages: initially, a base detector is trained using the duck dataset in the first phase; subsequently, in the second phase, the model undergoes fine-tuning to adapt and accurately identify the features of the new categories, chickens and fish. For each few-shot scenario, a small support set containing K randomly selected images (where K represents the number of shots, e.g., one-shot, two-shot) was assembled; each image in this support set depicted a single isolated object with no occlusion. All remaining images in this curated dataset then served as the query set to rigorously verify the model’s generalization capacity under practical evaluation conditions. This phased training approach is designed to enhance the model’s adaptability and accuracy in recognizing new categories. To demonstrate the stability of our method, each experiment was run three times using different seeds (2022–2024), and the mean and variance of the results were calculated.

In the image processing of the dataset, this study adopted a diversified input size strategy to enhance the generalization ability of the SSNFNet model. During the training phase, a range of minimum input sizes was utilized, including 480, 512, 544, 576, 608, 640, 672, 704, 736, 768, 800, etc. This multi-size input approach aids the model in effectively learning how to handle images of varying sizes. For testing, a consistent minimum input size of 800 was chosen to maintain uniformity in performance evaluation. Additionally, image cropping functionality was enabled to accommodate different sizes and proportions of images, ensuring the model’s excellent performance under various conditions. Through such a meticulous experimental setup, we aim to comprehensively evaluate the performance of the SSNFNet model in processing poultry datasets, especially those with significant differences in morphology, behavior, and habitat.

The experiments were conducted using the PyTorch 1.10.1 framework on a machine equipped with four NVIDIA RTX 2080 Ti GPUs (Santa Clara, CA, USA) (each with 11 GB of memory), an Intel(R) Xeon(R) Platinum 8255C CPU (Santa Clara, CA, USA) running at 2.50 GHz with 48 virtual cores, and 40 GB of system memory (RAM) (Intel, Santa Clara, CA, USA). It is important to note that the accuracy of few-shot learning can be significantly influenced by the number of GPUs used during training. All few-shot object detection models were trained and evaluated using the same four-GPU configuration to ensure the consistency, accuracy, and reliability of the experimental results.

In terms of training efficiency, each training epoch took approximately 10 s to complete, and the models were trained for a total of 200 epochs. As a result, the full training process for each model required no more than 30 min. For inference, the model achieved a speed of 4 s to process 126 images on an NVIDIA RTX 3060 GPU (Santa Clara, CA, USA), resulting in an approximate inference rate of 31 frames per second (FPS). We have further validated these results through preliminary deployment experiments on Jetson Nano hardware at 800 × 600 resolution, where the model maintained detection accuracy while achieving 8 FPS—comparable to FSCE (8 FPS) and superior to HTRPN (6 FPS) on identical hardware. This demonstrates our method’s practical deployment capability, as eight detections per second provides sufficient responsiveness for real-world object detection applications, while our careful design ensures no additional computational burden is introduced during inference compared to baseline methods. The original model has a size of 38.2 MB and requires approximately 4 GB of memory for inference. For deployment on edge devices, the model can be quantized, reducing its model size to around 19 MB and lowering its memory usage to approximately 2 GB, making it more suitable for resource-constrained environments.

To comprehensively evaluate the performance of the SSNFNet model in object detection tasks, we have chosen AP50 as our primary metric. AP50, a crucial indicator in the field of object detection, is primarily used to assess a model’s accuracy in detection. It focuses on measuring the degree of match between the model’s predicted bounding boxes and the actual bounding boxes, particularly when their Intersection over Union (IoU) reaches or exceeds 50%, at which point the prediction is considered correct. The calculation formula for the IoU is as follows:

(9) $$\mathrm{IoU}=\frac{\mathrm{Area}\;\mathrm{of}\;\mathrm{Intersection}}{\mathrm{Area}\;\mathrm{of}\;\mathrm{Union}}$$

here, “Area of Intersection” refers to the area of the overlap between the predicted and the actual bounding boxes, while “Area of Union” is the total area covered by both bounding boxes, which is the sum of their individual areas minus the area of intersection. The value of IoU ranges from 0 to 1, where 0 indicates no overlap and 1 indicates perfect overlap. This metric is extensively used to evaluate the performance of object detection models, especially their precision in detection.

The AP50 provides us with a quantitative measure of model performance relative a to higher IoU threshold under more lenient conditions, which helps assess the practicality and flexibility of the model in real-world applications. Especially in challenging few-shot learning scenarios, AP50 provides a clear standard to assess the model’s performance in terms of recognition accuracy. Moreover, due to its widespread application in the field of object detection, AP50 makes our research results comparably valuable and referential.

### 3.2. Comparative Experiments

We conducted a series of training and testing sessions on the poultry farming dataset using the popular few-shot object detection models: TFA w/cos [34], FSCE [26], Meta R-CNN [36], DeFRCN [37], and HTRPN [38]. The experimental results are shown in Table 1 and Table 2. Although there were cases where HTRPN surpassed FSCE in model efficacy, it was essential to recognize that the HTRPN’s performance was significantly influenced by the number of GPUs, leading to considerable fluctuations in accuracy. Given this observation, we ultimately chose the FSCE model as the basis for our research. The FSCE model demonstrated superior stability and accuracy, making it more suitable for our application needs in the poultry farming domain.

In our comprehensive evaluation of SSNFNet across diverse few-shot scenarios—spanning one-shot, two-shot, three-shot, and five-shot settings—SSNFNet has unequivocally set a new benchmark in the realm of object detection. Detailed in Table 1 and Table 2, these experiments demonstrate SSNFNet’s unparalleled performance, significantly surpassing that of existing models and cementing its status as the superior choice for nuanced detection tasks. A standout achievement of SSNFNet is its exceptional proficiency in the detection of aquatic species, such as fish, underscoring its versatility. Taking Table 2 as an example, within the three-shot paradigm, SSNFNet achieved groundbreaking detection accuracies (mAP50) of 82.75% for chickens and 66.00% for fish, culminating in an impressive average accuracy of 74.38%. Advancing to the five-shot scenario, SSNFNet further showcased its formidable capacity by elevating the average accuracy to an astounding 81.93%, a testament to its robustness and efficacy far beyond other few-shot detection models.

Beyond standard mAP50 comparisons, we conducted extensive evaluations to assess detection robustness under varying localization precision requirements. Specifically, we report AP scores at finer-grained IoU thresholds—ranging from AP50 to AP95—in Table 3. This in-depth analysis reveals that SSNFNet not only achieves superior performance at conventional thresholds, but also maintains strong accuracy even under stringent localization constraints (e.g., AP75 and AP95), highlighting its superior regression capability and detection stability. Moreover, to further validate the effectiveness of our method from different perspectives, we conducted additional evaluations on a challenging object detection scenario with severe occlusions (i.e., the Fish dataset), beyond the commonly used mAP metric. Specifically, we selected F1 Score, Recall, and Precision as key evaluation metrics to provide a more comprehensive understanding of detection performance. As shown in Table 4, all metrics demonstrate consistent improvement as the number of shots increases, indicating that our method offers stable and comprehensive enhancement in complex detection scenarios.

Moreover, a meticulous comparative analysis with well-known traditional object detection frameworks, including RetinaNet [39], CenterNet [40], FCOS [41], EfficientDet [42], YOLOv5 [43], YOLOv7 [44], and YOLOv8 [45], further highlights SSNFNet’s exceptional adaptability and performance. The results, presented in Table 1 and Table 2, affirm SSNFNet’s consistent dominance over both traditional and few-shot object detection models across all few-shot settings tested. This remarkable superiority is not merely a statistical triumph, but a beacon of SSNFNet’s practical applicability, especially within the agricultural domain, where acquiring extensive labeled datasets is often a prohibitive challenge. SSNFNet’s ability to deliver high accuracy with minimal training samples is not just innovative; it represents a paradigm shift in object detection, opening new vistas for efficient, scalable deployment in varied and resource-constrained environments.

**Figure 7:**
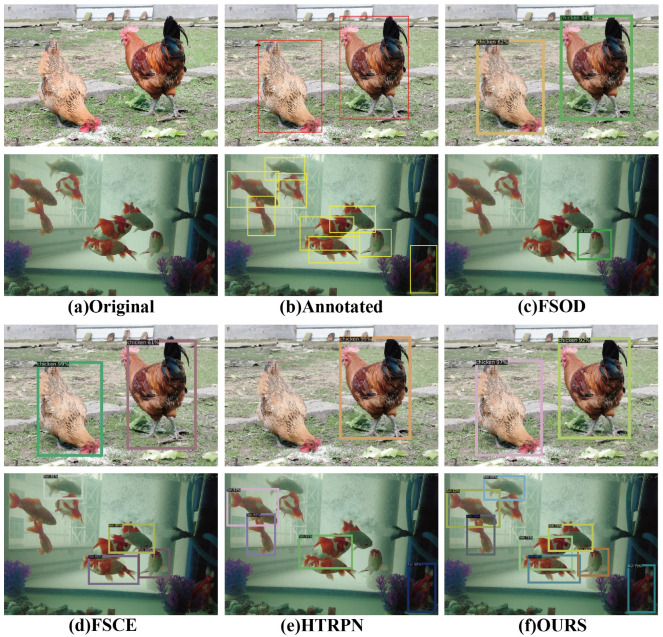
Taking five-shot as an example, the visualization results for chickens are shown: (**a**) the original image of the chickens, (**b**) the annotated image, and (**c**–**f**) the results from different models.

To vividly showcase the prowess of our SSNFNet model, we have utilized compelling visual representations, particularly emphasizing the five-shot scenario for its illustrative potential. As depicted in Figure 7, SSNFNet not only excels in the precision of detecting individual targets, but also demonstrates an exceptional capability to identify a higher number of objects within the same image. It is this blend of high precision and the capability to handle high-density object scenarios that underscores SSNFNet’s exceptional utility and positions it as a groundbreaking advancement in the field of object detection, particularly suited for applications demanding meticulous attention to detail and high fidelity in object recognition.

**Figure 8:**
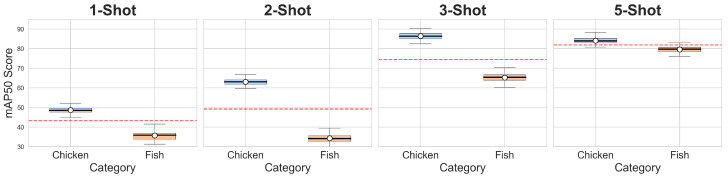
Performance stability analysis of our method across different random seeds (2021–2025). The boxplots demonstrate the distribution of mAP50 scores for chicken (blue) and fish (orange) categories under five-shot, two-shot, three-shot, and five-shot settings. The horizontal red dashed lines indicate the reference mean values for chicken and fish, respectively. The consistent performance across all configurations confirm the robustness of our method.

To further validate the stability of our method, we extended the experimental setup from three random seeds (2022–2024) to five seeds (2021–2025) and visualized the final results, as shown in Figure 8, demonstrating that our method maintains consistent performance in terms of both mean and variance metrics, which not only confirms its robustness, but also highlights its potential for reliable deployment in open-world scenarios.

**Figure 9:**
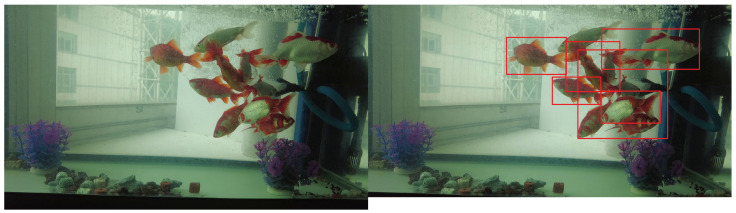
Examples of detection failures in challenging scenarios, demonstrating missed detections and incorrect grouping caused by dim lighting and severe occlusion.

We also analyzed some failure cases, as shown in Figure 9. We observed that under dim lighting and extreme occlusion conditions, the model tends to mislabel multiple overlapping fish as a single fish. Moreover, when a fish is extensively occluded, it is often missed by the model. These issues primarily explain why the model performs worse on the fish dataset compared to the chicken dataset.

### 3.3. Ablation Experiments

In the ablation study section of this paper, we systematically explore the effects of Soft-NMS and Sharpness-Aware Minimization (SAM) on enhancing the performance of our proposed SSNFNet model. All ablation experiments were conducted on a poultry breeding dataset under a five-shot setting, aiming to precisely evaluate the contributions of these techniques to the task of few-shot object detection.

We conducted four sets of experiments. Initially, the baseline FSCE model, which did not incorporate Soft-NMS or SAM, achieved an average accuracy of 73.66%. When Soft-NMS was introduced without the use of SAM, there was an increase in detection accuracy for chickens to 81.75%, while the accuracy for fish was 66.56%, resulting in an improved average accuracy of 74.16% compared to the baseline model. These results indicate that Soft-NMS can enhance the model’s poultry detection performance through its more nuanced handling of overlapping detections. Table 5 presents the results of our model ablation experiments.

The integration of SAM without Soft-NMS led to a notable improvement in fish detection accuracy, which increased to 72.90%, and chicken detection also improved to 85.52%. The average accuracy improved to 79.21%. This indicates that the SAM component significantly contributes to the model’s robustness, particularly in complex aquatic environments, where the background may introduce non-smooth loss challenges.

Our complete SSNFNet model, which incorporates both Soft-NMS and SAM, achieved the highest accuracies of 87.12% for chickens and 76.74% for fish, with an overall average accuracy of 81.93%. The combined effect of Soft-NMS and SAM in SSNFNet demonstrates a synergistic improvement, confirming that the integration of these two components can effectively enhance the model’s detection performance across different species in agricultural breeding scenarios.

### 3.4. Parameter Analysis

We also conducted parameter experiments, where a detailed analysis of parameters underscored the significance of fine-tuning hyperparameters for achieving optimal model performance. When testing one hyperparameter, all other hyperparameters were kept constant. By adjusting the rho value in SAM, the use of SAM-adaptive, the application of SAM-nesterov, the sigma value in Soft-NMS, and the score threshold of Soft-NMS, we observed significant differences in model performance across various parameter combinations.

The rho parameter in SAM dictates the model’s sensitivity to the local geometric properties of the loss function. A lower rho value results in a gentler adjustment of model sharpness, enhancing accuracy across different categories. The SAM-adaptive parameter determines whether the SAM optimizer adjusts its behavior adaptively to fit the data characteristics. In the SAM-nesterov setting, enabling nesterov-accelerated gradient (NAG) boosts the momentum of the optimization process, aiding the model in converging faster to superior solutions. Soft-NMS adjusts the strictness of non-maximum suppression (NMS) through the sigma parameter, mitigating penalties on highly overlapping boxes by modifying weights. The score threshold of Soft-NMS specifies the minimum score for retaining targets during the Soft-NMS process, with its adjustment balancing the quantity and quality of detection outcomes.

As shown in Table 6, the model performs optimally on datasets of chickens and fish when the SAM’s rho value is set to 0.01, SAM-adaptive is false, SAM-nesterov is true, the sigma value of Soft-NMS is 0.6, and the score threshold of Soft-NMS is 0.9, achieving an average accuracy of 81.93%. It is noted that the optimal settings for these parameters may vary across different datasets.

## 4. Discussion

### 4.1. Contribution to Intelligent Poultry Farming

This study introduces a specialized few-shot object detection model called SSNFNet for the poultry farming sector, achieving significant results. This approach allows for accurate identification and tracking of poultry, even with limited data samples, substantially improving the capability of farms to diagnose diseases early, monitor for abnormal behaviors, and optimize production efficiency. Automated monitoring systems enable real-time observation of poultry health and behavioral patterns, facilitating prompt detection and response to health issues, minimizing disease spread and impact. Moreover, the application of this technology aids in refining feeding strategies and living conditions, enhancing resource utilization, reducing waste, and ultimately increasing the overall productivity of poultry farming. This research not only provides an innovative method for intelligent poultry cultivation, but also offers valuable insights for practices in precision agriculture.

### 4.2. Comparison of Methods

In the domain of poultry farming, traditional object detection technologies face significant challenges due to their reliance on extensive annotated datasets for training models to achieve desired accuracy and generalization capabilities. This process often demands the collection and annotation of thousands of images, covering a variety of poultry species, behaviors, and potential farming environmental conditions, which introduces considerable upfront costs and time investment. However, the introduction of SSNFNet—a breakthrough based on few-shot object detection technology—marks a significant advancement. SSNFNet dramatically reduces the dependency on extensive annotated data, enabling the recognition of new species or behaviors with training on just a handful of images. This not only accelerates the deployment of models significantly, but also reduces costs markedly. Importantly, it elegantly addresses the complexity and variability of poultry farming environments, offering a more flexible and efficient solution.

Contrastingly, traditional object detection methods, when confronted with new scenarios or environmental changes, might necessitate the re-collection of extensive data and re-training of the model, a process that is both time-consuming and costly. In stark contrast, SSNFNet can quickly learn and adapt based on a few examples. This flexibility and robust adaptability allow for SSNFNet to swiftly accommodate new poultry breeds or previously un-encountered specific behavior patterns, demonstrating superior generalization capabilities. In this manner, SSNFNet contributes a reliable tool for intelligent poultry farming, providing ongoing monitoring and management in the ever-changing farming environment.

This contribution not only signifies technological progress, but also paves the way for new possibilities in the digital transformation of the poultry farming industry, establishing a robust foundation for future advancements. Through diminishing reliance on extensive datasets, the implementation of SSNFNet accelerates innovation and provides substantial support for animal welfare and disease management.

### 4.3. Limitations and Future Developments

Our study has made certain progress in exploring the application of few-shot object detection in the field of poultry farming, yet there are still some limitations, which also point the way for future research.

Firstly, regarding the generalizability and dataset scope of SSNFNet, it is pertinent to highlight that our current dataset, while centered on chickens, ducks, and goldfish, encompasses notable environmental and acquisitional variability. Specifically, data was gathered under diverse lighting conditions resulting from collection at different times of day, utilizing various acquisition devices which introduced sensor noise, and spanning distinct farming environments—for example, chicken data was sourced from three separate farms featuring different housing conditions. The inclusion of these variations allowed for our experiments to assess the model’s capability in extracting robust deep semantic features, a critical aspect of effective few-shot learning. Indeed, SSNFNet demonstrated commendable robustness in handling these inherent dataset variations. However, we acknowledge that the geographic spread of our data collection sites and the diversity of specific environmental factors (e.g., unique housing structures not encountered, extreme weather conditions, or a vastly wider range of animal species) are not exhaustive. This could introduce biases if the model is deployed in regions or conditions starkly different from those in our training and testing data, potentially limiting its out-of-the-box generalizability to such novel settings. Investigating the model’s operational boundaries under such increasingly complex and varied scenarios will be a key focus of subsequent work.

Secondly, real-world deployment of SSNFNet in operational farm settings presents practical constraints that were not exhaustively addressed in this study. For instance, variations in sensor types, quality, age, and calibration across different farms, or even over time on the same farm, can lead to inconsistencies in input data, potentially affecting model performance. More critically, occlusion issues, where animals are partially or fully hidden by other animals, feeding troughs, drinkers, or structural elements of their enclosures, are frequent occurrences in densely populated poultry environments. Such occlusions can significantly impair the model’s detection accuracy and reliability. Future work should explicitly investigate strategies to enhance model robustness against these sensor variations and develop more sophisticated methods for handling occluded objects in real-world agricultural settings.

Thirdly, our proposed SSNFNet model adopts a two-stage training approach, where the first stage relies on a larger initial dataset to train the foundational network. This reliance limits the model’s adaptability in extreme few-shot scenarios where such initial large datasets are unavailable. Moving forward, we will explore more efficient few-shot learning strategies to reduce dependence on large-scale initial datasets, potentially investigating one-stage architectures or meta-learning approaches that are inherently more data-efficient.

Fourthly, the performance of the model is to some extent affected by computational resources, especially the number of GPUs available. We have observed a significant improvement in the model’s performance with an increase in the number of available GPUs. This phenomenon suggests that, despite the fact that the model’s design considers computational efficiency, its performance advantage might be diminished in resource-constrained situations, such as on lower-resource farms or edge computing devices. In response to the relationship between model performance and computational resources, future work will focus on optimizing the model’s structure, exploring model compression techniques, and refining the training process to achieve high performance even with limited computational resources, thereby enhancing its accessibility and applicability in diverse farming contexts.

In summary, the journey ahead remains full of opportunities for innovation. Despite the constraints brought about by data scarcity, deployment complexities, and limited computational resources, which test the resilience of our methodologies, they also inspire creative solutions and development strategies. As we progress, the integration of more effective learning methods and the optimization of model architectures hold the promise of ushering in a new era of precision and flexibility in agricultural technology.

## 5. Conclusions

In conclusion, SSNFNet introduces a groundbreaking few-shot object detection method to smart agriculture, significantly enhancing animal monitoring with minimal data requirements. By overcoming the limitations of traditional detection methods and reducing reliance on extensive annotations, SSNFNet promotes scalable and efficient agricultural practices. Based on the FSCE framework and enriched with SAM optimization, SSNFNet mitigates the model’s tendency to overfit specific training samples, thereby boosting its generalization capabilities. The implementation of Soft-NMS ingeniously addresses the issue of overlaps, alleviating the decrease in detection accuracy in scenarios of animal congregation. SSNFNet’s exceptional performance in detecting a variety of animal species, and its successful extension to aquatic organisms like fish, underscores its robustness and potential for broader application.

For future research, we plan to further refine the proposed network structure, delving into optimizations that balance precision and speed to achieve high-accuracy real-time object detection. This pursuit will include exploring enhancements to the model’s adaptability and efficiency, ensuring that it can meet the evolving demands of smart agriculture and, potentially, beyond.

## Figures and Tables

**Table 1:** One-shot and two-shot detection performance (mAP50) on the poultry farming dataset. The Average results are colered gray. The best results are bolded.

Method		One-Shot			Two-Shot		
		Chicken	Fish	Avg	Chicken	Fish	Avg
Traditional	RetinaNet	9.10±1.04	0±0	4.55	3.06±0.65	0±0	1.53
	CenterNet	0.03±0.01	0±0	0.02	12.88±1.80	1.24±0.35	7.06
	FCOS	5.41±1.93	0.04±0	2.73	5.63±0.90	0±0	2.82
	EfficientDet	0.86±0.16	0.01±0	0.44	1.09±0.25	0±0	0.55
	YOLOv5	6.08±0.85	9.68±0.70	7.88	3.56±0.45	7.79±0.73	5.68
	YOLOv7	1.10±0.20	5.34±0.95	3.22	2.04±0.30	4.11±1.66	3.08
	YOLOv8	0.70±0.15	0.03±0	0.37	2.00±0.25	0.28±0.05	1.14
Few-Shot	TFA w/cos	68.35±2.10	13.37±1.55	40.86	53.38±2.80	9.09±0.88	31.24
	FSCE	33.65±2.30	36.71±2.50	35.18	53.64±1.90	42.98±2.60	48.31
	Meta R-CNN	31.03±3.18	33.61±1.44	32.32	54.25±0.67	40.84±3.30	47.55
	DeFRCN	36.22±1.17	36.71±2.70	35.18	61.15±5.02	42.98±2.90	52.07
	HTRPN	40.29±1.85	35.43±2.15	37.86	56.84±2.55	40.51±2.85	48.68
	SSNFNet (Ours)	49.48±1.95	36.87±2.25	**43.18**	63.82±1.70	34.52±2.35	**49.17**

**Table 2:** Three-shot and five-shot detection performance (mAP50) on the poultry farming dataset. The Average results are colered gray. The best results are bolded.

Method		Three-Shot			Five-Shot		
		Chicken	Fish	Avg	Chicken	Fish	Avg
Traditional	RetinaNet	4.96±0.72	3.93±0.58	4.45	15.39±2.05	0.19±0.03	7.79
	CenterNet	15.40±1.98	0.45±0.10	7.93	19.10±2.45	2.23±0.38	10.67
	FCOS	11.86±1.65	3.38±0.55	7.62	29.65±2.75	0.65±0.12	15.15
	EfficientDet	2.65±0.40	0±0	1.33	10.35±1.30	0.17±0.02	5.26
	YOLOv5	6.81±0.92	16.06±1.88	11.44	16.68±2.12	7.84±0.68	12.26
	YOLOv7	7.15±0.80	7.39±0.85	7.27	4.78±0.70	4.75±0.65	4.77
	YOLOv8	5.04±0.78	0.07±0.01	2.56	5.49±0.82	5.47±0.80	5.48
Few-Shot	TFA w/cos	72.73±2.30	45.16±2.60	58.95	81.82±1.90	54.07±2.20	67.95
	FSCE	74.57±1.80	60.49±2.10	67.53	77.56±2.40	69.75±2.70	73.66
	Meta R-CNN	78.12±2.36	51.69±1.94	64.91	78.10±2.58	68.08±5.39	73.09
	DeFRCN	76.65±4.41	63.02±3.20	69.84	86.07±3.89	66.24±4.23	76.16
	HTRPN	71.68±2.00	61.54±2.30	66.61	86.18±1.85	69.82±2.15	78.00
	SSNFNet(Ours)	82.75±1.75	66.00±2.05	**74.38**	87.12±1.60	76.74±1.90	**81.93**

**Table 3:** Few-shot detection performance on the poultry farming dataset (chicken), with AP scores at different IoU thresholds. The best results are bolded.

Shot	Model Name	AP50	AP60	AP70	AP75	AP80	AP90	AP95
		(%)	(%)	(%)	(%)	(%)	(%)	(%)
1	TFA w/cos	**68.35**	**45.21**	38.37	**34.12**	28.12	10.34	6.21
	FSCE	33.65	29.47	24.56	20.12	17.89	7.01	2.10
	Meta R-CNN	31.03	27.12	22.34	18.01	15.78	6.23	1.89
	DeFRCN	36.22	31.45	26.01	21.34	18.89	7.67	2.34
	HTRPN	40.29	35.21	29.12	24.23	20.45	8.98	2.87
	SSNFNet (Ours)	49.48	45.12	**40.23**	**34.12**	**30.45**	**18.12**	**7.34**
2	TFA w/cos	53.38	48.23	40.12	34.15	30.14	15.89	5.23
	FSCE	53.64	47.56	40.47	34.01	31.01	16.45	5.78
	Meta R-CNN	54.25	48.01	41.23	35.01	32.12	17.01	6.01
	DeFRCN	61.15	54.01	46.23	40.12	35.67	18.89	6.23
	HTRPN	56.84	50.12	42.56	37.23	33.12	17.65	6.12
	SSNFNet (Ours)	**63.82**	**57.34**	**49.65**	**44.32**	**39.23**	**21.45**	**8.01**
3	TFA w/cos	72.73	66.12	56.78	50.12	43.34	19.45	6.32
	FSCE	74.57	67.34	58.65	52.11	46.12	20.12	6.78
	Meta R-CNN	78.12	70.34	61.23	54.32	48.78	21.34	7.01
	DeFRCN	76.65	69.23	59.87	53.45	47.01	20.98	6.98
	HTRPN	71.68	64.32	55.34	49.21	43.67	19.23	6.45
	SSNFNet (Ours)	**82.75**	**74.12**	**64.34**	**58.23**	**51.23**	**24.10**	**9.12**
5	TFA w/cos	81.82	74.23	64.12	58.34	50.78	22.45	7.34
	FSCE	77.56	69.78	60.45	54.21	48.12	21.01	7.01
	Meta R-CNN	78.10	70.01	61.12	55.32	49.01	22.34	7.56
	DeFRCN	86.07	78.34	68.67	63.21	54.89	24.12	8.01
	HTRPN	86.18	78.89	69.01	63.45	55.23	24.78	8.23
	SSNFNet (Ours)	**87.12**	**79.01**	**69.12**	**64.01**	**56.65**	**26.34**	**9.32**

**Table 4:** F1 Score, Recall, and Precision on the fish dataset under different shot settings.

Shot	Fish AP (%)	F1 Score	Recall (%)	Precision (%)
One-shot	31.76	0.35	77.39	22.38
Two-shot	53.92	0.41	86.51	26.46
Three-shot	67.65	0.49	87.97	34.06
Five-shot	75.77	0.62	87.14	48.44

**Table 5:** Ablation for key components proposed in SSNFNet.

Model	Soft-NMS	SAM	Chicken	Fish	Avg
Baseline	✘	✘	77.56	69.75	73.66
	✔	✘	81.75	66.56	74.16
	✘	✔	85.52	72.90	79.21
SSNFNet (Ours)	✔	✔	87.12	76.74	81.93

**Table 6:** Results of the parameter analysis conducted on the poultry farming dataset.

Settings	Value	Chicken	Fish	Avg
SAM-rho	0.01	87.12	76.74	81.93
	0.05	83.87	70.46	77.17
	0.1	82.85	65.53	74.19
SAM-adaptive	False	87.12	76.74	81.93
	True	87.70	60.04	73.87
SAM-nesterov	False	83.41	71.17	77.29
	True	87.12	76.74	81.93
Soft-NMS-σ	0.4	79.22	55.91	67.57
	0.5	88.86	72.67	80.77
	0.6	87.12	76.74	81.93
Soft-NMS-Score threshold	0.80	89.64	59.57	74.61
	0.90	87.12	76.74	81.93
	0.95	85.59	64.71	75.15

## Data Availability

The dataset used in this study was collected and manually annotated by our research team. The dataset is accessible at google drive (https://drive.google.com/file/d/1KQQl3afV8TJ7lf_4gaFK4xk0hzyCPDTr/view?usp=drive_link) (accessed on 29 July 2025).
